# 328. Universal PCR Testing- Is It Worth It?

**DOI:** 10.1093/ofid/ofac492.406

**Published:** 2022-12-15

**Authors:** Navina K Birk, Kasturi Saikia, Mayur Ramesh, Linoj Samuel, Robert Tibbetts

**Affiliations:** Henry Ford Hospital, Detroit, Michigan; Henry Ford Hospital, Detroit, Michigan; Henry Ford Hospital, Detroit, Michigan; Henry Ford Hospital, Detroit, Michigan; Henry Ford Health, Detroit, Michigan

## Abstract

**Background:**

Universal PCR and Next Generation Sequencing (uPCR/NGS) is a major advancement in microbiology. It is a highly sensitive and specific test that amplifies ribosomal RNA in samples to detect bacterial and fungal pathogens. We investigated the uPCR/NGS tests sent from Henry Ford Health (HFH) and the effect obtaining this test had on patient care.

**Methods:**

We completed a retrospective, observational study assessing all consecutive uPCR/NGS tests obtained from at HFH from 2016-2021. This included uPCR/NGS for detection of bacterial, fungal, mycobacterium tuberculosis (MTB), and non-tuberculosis mycobacterium (NTM). All samples were of non-blood fluids and tissue samples. Patients concurrent tissue cultures and blood cultures from day of uPCR/NGS, and within 6 months of obtaining the uPCR/NGS sample were evaluated. Primary outcomes included if uPCR/NGS testing resulted in a change of choice or duration of antibiotic therapy.

**Results:**

At HFH, 226 uPCR/NGS tests from 111 samples were analyzed. This included 83 bacterial, 51 MTB, 51 NTM, and 41 fungal uPCR/NGS tests. Of the 226 uPCR/NGS tests, 31 tests (13.7%) resulted with positive result on uPCR/NGS. A total of 31 tests (13.7%) were ordered on a known culture positive sample, and 195 (86.3%) were ordered on a culture negative sample.

Of samples sent from a culture positive sample, 7 (22.6%) resulted with positive uPCR/NGS results. 3 (42.9%) matched the tissue cultures, and 2 (28.6%) matched the patients’ blood cultures. None of the patients had any change in choice or duration of antibiotics based on uPCR/NGS results.

Of the samples sent from a culture negative sample, 24 (12.3%) samples resulted with positive uPCR/NGS results. 2 (8.3%) matched a patient’s tissue cultures and none matched blood cultures. Change in antibiotic choice or duration occurred in 16 (17.6%) patients. The samples with the highest rates of change in management included cerebrospinal fluid (42.9%), brain tissue (36.4%), cardiac valvular tissue (27.3%), lymph nodes (25%), synovial fluid (20%).

**Figure 1:**
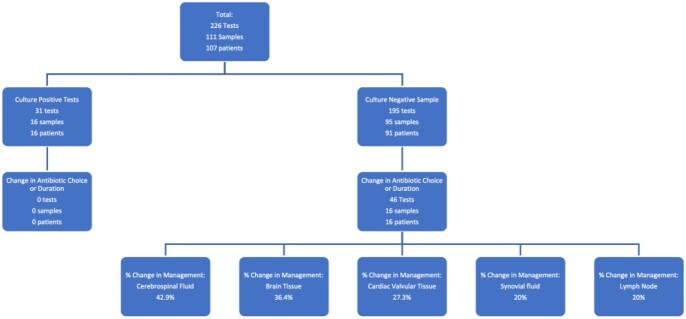
Flowchart of Results and Impact on Management

**Conclusion:**

Our results show that uPCR/NGS has an overall low percent of impact in clinical outcomes, but it is a useful test in when used on a culture negative sample in certain sample types, along with a high index of suspicion for infection.

**Disclosures:**

**All Authors**: No reported disclosures.

